# Erythrocyte Ascorbate Is a Potential Indicator of Steady-State Plasma Ascorbate Concentrations in Healthy Non-Fasting Individuals

**DOI:** 10.3390/nu12020418

**Published:** 2020-02-06

**Authors:** Juliet M. Pullar, Susannah Dunham, Gabi U. Dachs, Margreet C. M. Vissers, Anitra C. Carr

**Affiliations:** 1 Centre for Free Radical Research, Department of Pathology and Biomedical Science, University of Otago, Christchurch, PO Box 4345, 8140 Christchurch, New Zealand; Susannah.Dunham@cdhb.health.nz (S.D.); margreet.vissers@otago.ac.nz (M.C.M.V.); 2 Mackenzie Cancer Research Group, Department of Pathology and Biomedical Science, University of Otago, Christchurch, PO Box 4345, 8140 Christchurch, New Zealand; gabi.dachs@otago.ac.nz; 3 Nutrition in Medicine Research Group, Department of Pathology and Biomedical Science, University of Otago, Christchurch, PO Box 4345, 8140 Christchurch, New Zealand; anitra.carr@otago.ac.nz

**Keywords:** vitamin C, ascorbate, plasma, erythrocyte, steady-state, pharmacokinetic, dehydroascorbic acid

## Abstract

Plasma vitamin C concentrations fluctuate in response to recent dietary intake; therefore levels are typically determined in the fasting state. Erythrocyte ascorbate concentrations have been shown to be similar to plasma levels, but little is known about the kinetics of ascorbate accumulation in these cells. In this study, we investigated ascorbate uptake into erythrocytes after dietary supplementation with vitamin C and compared it to changes in plasma ascorbate concentrations. Seven individuals with baseline fasting plasma vitamin C concentrations ≥ 50 µmol/L were depleted of vitamin C-containing foods and drinks for one week, and then supplemented with 250 mg vitamin C/day in addition to resuming their normal diet. Fasting or steady-state plasma ascorbate concentrations declined to almost half of their baseline concentration over the week of vitamin C depletion, and then returned to saturation within two days of beginning supplementation. Erythrocyte ascorbate concentrations exhibited a very similar profile to plasma levels, with values ~76% of plasma, and a strong linear correlation (r = 0.89, *p* < 0.0001). Using a pharmacokinetic study design in six individuals with baseline fasting plasma vitamin C concentrations ≥50 µmol/L, we also showed that, unlike plasma, which peaked between 2 and 4 h following ingestion of 200 mg of vitamin C, erythrocyte ascorbate concentrations did not change in the six hours after supplementation. The data from these two intervention studies indicate that erythrocyte ascorbate concentration provides a stable measure of steady-state plasma ascorbate status and could be used to monitor ascorbate status in healthy non-fasting individuals.

## 1. Introduction

Humans, unlike most other animals, have lost the ability to synthesise vitamin C (ascorbate) due to evolutionary conserved mutations in the gene encoding L-gulonolactone oxidase, which catalyses the final step in the biosynthetic pathway [1]. A diet severely lacking in vitamin C can result in the deficiency disease scurvy, which is characterised by the breakdown of connective tissue, causing localised bruising and bleeding, and ultimately leading to death [2,3]. Although scurvy is now rarely seen, the inadequate dietary intake of vitamin C is thought to be much more common and hypovitaminosis C (plasma ascorbate concentrations < 23 µmol/L) has been described in up to 15% of individuals [4,5]. These levels are associated with early symptoms of scurvy, such as fatigue and depression [6].

After ingestion, vitamin C is absorbed via the small intestine, released into the bloodstream and distributed to the tissues [7]. Accumulation into cells occurs via the sodium-dependent vitamin C transporters SVCT1 and SVCT2, which actively transport the vitamin against a concentration gradient to reach millimolar concentrations inside the cells [8,9]. It is well recognised that the SVCTs are vital for ascorbate distribution in the body [10,11]. Cells can also transport the oxidised form of vitamin C, dehydroascorbic acid (DHA) using the facilitative glucose transporters (GLUTs) in competition with glucose [12,13]. Once inside the cells, DHA is reduced to ascorbate [9,14]. GLUT-mediated DHA transport seems to be critical for ascorbate uptake into the erythrocytes. Mature erythrocytes do not contain SVCT proteins [15,16] and are thought to be reliant on the GLUTs, particularly GLUT1, for obtaining ascorbate from circulation [13,17,18]. Previous studies have shown that the ascorbate concentration in erythrocytes is similar to that of plasma [19,20,21], indicating that these cells do not concentrate ascorbate against the plasma concentration gradient.

Vitamin C status is typically determined by measuring the plasma concentrations of the vitamin [5,14]. However, the accuracy of this measurement is dependent on the use of fasting blood samples, as plasma vitamin C levels can fluctuate depending on recent dietary intake [15]. Providing fasting samples can be clinically challenging and inconvenient, and can also be difficult to incorporate into some study design scenarios. Thus, an accurate measurement of the vitamin C status in non-fasting individuals would be a useful tool. Given the observation that erythrocyte ascorbate levels are similar to plasma levels [19,20,21], we hypothesised that the intracellular erythrocyte ascorbate concentration may be a useful indicator of the steady-state plasma ascorbate concentration. In this study, we used both steady-state and short-term pharmacokinetic study designs to determine the kinetics of ascorbate uptake into erythrocytes and the relationship between erythrocyte levels and plasma ascorbate status.

## 2. Materials and Methods

### 2.1. Steady-State Study

**Figure 1:**
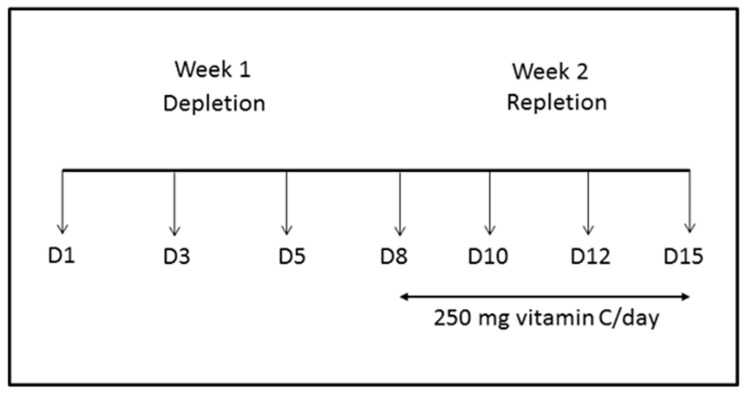
Steady-state study design. Participants were depleted of vitamin C-containing foods and beverages for one week and then supplemented with 250 mg of vitamin C per day for another week, in addition to returning to their normal diet. Fasting blood samples were obtained at the days indicated and analysed for plasma and erythrocyte ascorbate (D1 is day 1, etc.).

Ethical approval was obtained from the New Zealand Southern Health and Disability Ethics Committee URA/06/12/083/AM02. Seven healthy participants were recruited from the University of Otago, Christchurch, with all participants providing written informed consent. Participants were eligible if they were non-smokers and had fasting plasma ascorbate concentrations of ≥50 µmol/L. The study design is shown in Figure 1. At recruitment, participants were asked to refrain from eating and drinking vitamin C-containing food for one week and were provided with an extensive list of foods to avoid, as well as a list of those that lacked vitamin C. After one week, participants resumed their normal diet and were given a 250 mg vitamin C tablet daily (Tishcon Corp., Westbury, NY, USA). Plasma and erythrocyte ascorbate concentrations were assessed at day 1 and every 2–3 days throughout the study period. All blood samples were obtained via venous puncture after an overnight fast, which included sampling prior to the ingestion of the daily vitamin C supplement in the second week of the study.

### 2.2. Pharmacokinetic Study

Ethical approval was obtained from the University of Otago Ethics Committee (H14/123) to conduct a short-term pharmacokinetic study, comparing the uptake of ascorbate into plasma and erythrocytes following dietary intake. Six healthy volunteers with fasting plasma ascorbate concentrations of ≥50 µmol/L were recruited from the University of Otago, Christchurch. Participants with healthy vitamin C levels (≥50 µmol/L) were chosen to avoid the possible preferential uptake of ascorbate by the tissues in individuals with a low vitamin C status, which could confound the comparison between plasma and erythrocyte ascorbate. All participants provided written informed consent. Following an overnight fast, a blood sample was obtained and the participants were supplemented with 200 mg of vitamin C tablets (Tishcon Corp., Westbury, NY, USA). Blood samples were collected every two hours for the next six hours (four samples in total, including the baseline). Following baseline blood collection, the participants were asked to avoid vitamin C-containing goods for the remaining six hours. The blood samples were processed for plasma and erythrocyte ascorbate analyses at the time of collection.

### 2.3. Plasma Ascorbate Sample Processing

Blood was collected in K_3_-EDTA vacutainer tubes (Becton Dickinson, Auckland, New Zealand), immediately placed on ice, and processed within two hours of collection. All the following procedures were carried out on ice or at 4 °C. The blood was centrifuged at 3200× *g* for 15 min at 4 °C to separate the cells from the plasma. An aliquot of plasma was removed for ascorbate analysis and was acidified with an equal volume of ice-cold 0.54 mol/L perchloric acid containing 100 µmol/L diethylenetriaminepentaacetic acid (DTPA). The perchloric acid extracts were centrifuged and supernatants stored at −80 °C until HPLC analysis.

### 2.4. Erythrocyte Ascorbate Sample Processing

An analysis of erythrocyte ascorbate was undertaken using an adaptation of the method of Levine and co-workers [21]. Firstly, the remaining plasma and buffy coat layer were carefully removed from the centrifuged blood and discarded. The packed erythrocytes were washed once with a 10-fold excess of ice-cold PBS containing 500 µmol/L DTPA. Following centrifugation, 150 µL aliquots of packed erythrocytes were stored at −80 °C. On the day of the HPLC analysis, the erythrocytes were rapidly thawed and the cells lysed with the addition of a four times volume of ice-cold milliQ water containing 500 µmol/L DTPA, vortex mixing and incubation on ice for 2 min. A 200 µL aliquot was added to the top of a centrifugal filter unit (Amicon Ultra 0.5 mL, 10K Ultracel^®^; Millipore) and the lysate was centrifuged at 14,000 × g for 20 min at 4 °C to remove haemoglobin. An equal volume of ice-cold 0.54 mol/L perchloric acid containing 100 µmol/L DTPA was immediately added to the ultrafiltrate and the samples were vortexed and spun. The samples were incubated with tris (2-carboxyethyl) phosphine hydrochloride (TCEP) to reduce any DHA present in the sample, as described previously [22].

### 2.5. Ascorbate HPLC Analysis

The vitamin C content of the plasma and erythrocyte samples was analysed by reverse-phase HPLC with coulometric electrochemical detection [22]. A standard curve of sodium ascorbate was freshly prepared each day in 77 mmol/L perchloric acid containing 100 µmol/L DTPA. Plasma and erythrocyte ascorbate are expressed as µmol/L.

### 2.6. Statistical Analyses

Statistical analyses were carried out using GraphPad Prism version 8 (La Jolla, CA, USA). The data are represented as the mean ± SEM, with *p* values ≤ 0.05 considered significant. Correlations were tested using Pearson’s linear correlation and differences between paired data were tested using two-tailed paired t-tests.

## 3. Results

### 3.1. Steady-State Study

**Figure 2:**
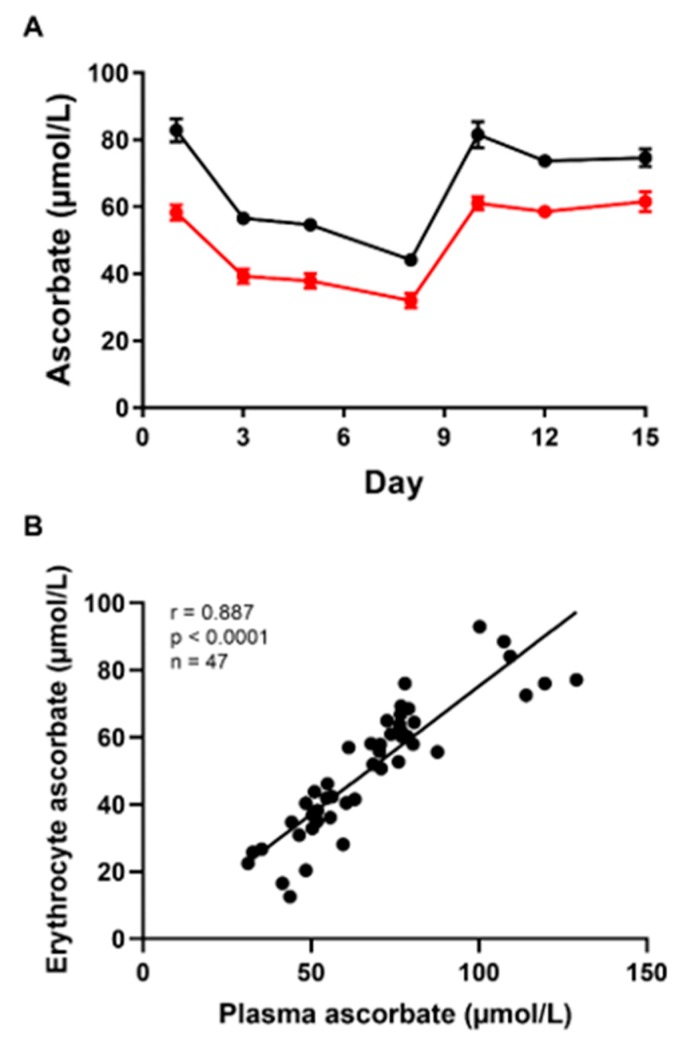
Changes in steady-state ascorbate concentrations during the two-week study. (**A**) Fasting plasma (●) and erythrocyte (●) ascorbate concentrations over time. Each symbol represents the mean ± SEM of 5to 7 individuals, as not all 7 individuals provided samples on each day of the study. (**B**) Linear correlation of the plasma and erythrocyte ascorbate concentrations (n = 47 points). For Figure A, paired t-tests showed that days 3, 5 and 8 were significantly different to the baseline for both plasma and erythrocytes. For Figure B, a Pearson linear correlation analysis was performed.

Seven healthy individuals with plasma vitamin C concentrations of ≥50 µmol/L were depleted via elimination of vitamin C-containing foods and drinks for one week and then supplemented with 250 mg of vitamin C/day for the following week, in addition to resuming their normal diet. Fasting plasma and erythrocyte ascorbate concentrations were measured every two to three days. The mean baseline fasting plasma ascorbate concentration was 82.8 ± 9.0 µmol/L (Figure 2A). Plasma ascorbate levels decreased rapidly upon withdrawal of vitamin C from the diet, reaching 44.1 ± 5.2 µmol/L after 7 days (Figure 2A; ~47% reduction). However, ascorbate concentrations were quickly restored upon the addition of vitamin C to the diet, with the group reaching plasma saturation (≥80 µmol/L) within two days of beginning supplementation.

Similarly, erythrocyte ascorbate concentrations decreased over the week in which vitamin C was removed from the diet, dropping from a starting concentration of 58.3 ± 6.0 µmol/L to 32.0 ± 5.7 µmol/L over the 7 days (Figure 2A; ~45% reduction). When participants were supplemented with vitamin C, a rapid and significant increase in erythrocyte ascorbate was observed. Like the plasma, the erythrocyte ascorbate concentration plateaued within two days of beginning supplementation.

The data show that changes in the steady-state ascorbate concentration of individuals, as evidenced by their fasting plasma ascorbate levels, are also reflected in their erythrocyte ascorbate content. Indeed, a strong positive linear relationship was observed between the two (Figure 2B: Pearson correlation coefficient r of 0.887; *p* < 0.0001). For every 1 µmol/L increase in plasma ascorbate, there was a ~0.76 µmol/L increase in erythrocyte ascorbate (regression line: y = 0.76 x −1), demonstrating a lower ascorbate concentration in the erythrocytes than in plasma. As expected, erythrocytes do not accumulate ascorbate to the millimolar concentrations observed in other cell types.

### 3.2. Short-Term Pharmacokinetic Study

**Figure 3:**
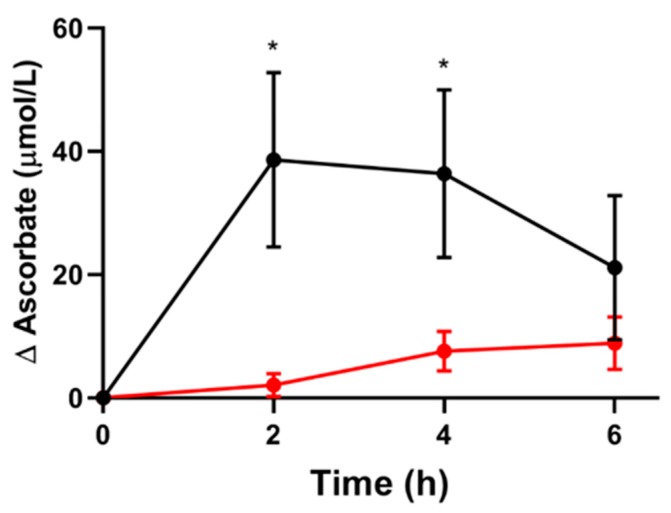
Pharmacokinetic study. The change in plasma (●) and erythrocyte (●) ascorbate following ingestion of 200 mg of vitamin C. The zero time point is fasting, with supplementation occurring immediately after this sample was taken. The data represent the mean ± SEM (n = 6). The baseline ascorbate concentrations were 67.2 ± 8.8 µmol/L and 58.7 ± 4.6 µmol/L for plasma and erythrocytes, respectively. Paired t-tests indicate that erythrocyte ascorbate time points were not significantly different from the baseline, whereas plasma ascorbate was different from the baseline at 2 and 4 h (*p* < 0.05). Furthermore, erythrocyte and plasma ascorbate were significantly different from each other at the time points indicated * (*p* < 0.02).

To investigate the short-term effects of vitamin C supplementation on erythrocyte ascorbate concentrations, a pharmacokinetic study was conducted in six individuals who had plasma vitamin C concentrations of ≥50 µmol/L. Fasting participants were given 200 mg vitamin C tablets and plasma and erythrocyte ascorbate were monitored over the following 6 h (Figure 3). A statistically significant increase in plasma ascorbate was observed at 2 and 4 h post-supplementation, with levels returning towards baseline at 6 h. In comparison, erythrocyte ascorbate did not significantly differ from baseline for the duration of the time course. A significant difference between plasma and erythrocyte ascorbate was found at two and four hours, but not at six 6 hours (*p* < 0.05). As such, plasma and erythrocyte ascorbate were not linearly correlated (r = 0.08, *p* = 0.7). The data indicate that erythrocyte ascorbate concentrations do not show the same rapid increase as occurs in plasma after the ingestion of vitamin C [6,23], suggesting that these levels more accurately reflect steady-state ascorbate levels and may be a useful indicator of their status in non-fasting blood samples.

## 4. Discussion

Unlike most other cell types, which actively take up ascorbate using the SVCTs, erythrocytes accumulate ascorbate primarily by the passive transport of DHA via the GLUTs [17]. In our study of healthy individuals, we found that the ascorbate content of erythrocytes did not change following dietary intake of the vitamin, despite there being a transient peak observed in the plasma. This indicates that erythrocyte ascorbate does not respond to a transient change in plasma levels and more accurately reflects steady-state plasma ascorbate. This was supported by the observation that changes in the steady-state plasma ascorbate concentration were reflected in the erythrocyte ascorbate concentration. Red cell ascorbate concentrations were ~0.76 of those of plasma, using the gradient of the regression line. These findings suggest that erythrocyte ascorbate content could be used as an indicator of the steady-state plasma ascorbate concentrations in non-fasting individuals, as this measurement does not seem to be subject to transient fluctuations following dietary intake.

It is well known that intracellular erythrocyte ascorbate concentrations are comparable to the plasma concentrations of the vitamin [14,19,20,21,24]; however, less is known about the short-term uptake kinetics of these cells. A previous study by Williamson and Winterbourn [19] indicated that plasma ascorbate concentrations increased two to three-fold two hours after oral administration of 2 g of vitamin C, whereas the erythrocyte ascorbate increased by only about 20%. Although this was higher than the increase observed in our study, it is likely to reflect the 10-fold higher vitamin C dose used in their study. They also conducted a supplementation study with 1 g of vitamin C/day for 2–3 weeks in 11 individuals and showed increases in both the fasting plasma and erythrocyte ascorbate concentrations. However, the ratio of erythrocyte to plasma ascorbate was ~1.6 in their study [19], which is higher than our measurements. This difference may relate to the colourimetric assay used to measure erythrocyte ascorbate in their study, which may be affected by haemoglobin iron and therefore, may be less accurate than our assay, which overcomes many of the problems associated with measuring erythrocyte ascorbate, notably the high iron content of these cells [21].

There are, however, some limitations to using erythrocyte ascorbate concentration as an indicator of steady-state plasma ascorbate status in non-fasting individuals. The erythrocyte assay is more expensive and time-consuming, with additional handling required over the plasma method [21]. Moreover, the values obtained cannot be used interchangeably with those of plasma. While we have found that erythrocyte ascorbate is about 75% of plasma ascorbate in our healthy cohort, and others have found a similar result [21], whether this ratio would hold for ascorbate concentrations above or below the normal range (~40–120 µmol/L in our study) or in individuals who are unwell and may have higher DHA concentrations due to oxidative stress is not clear. Recent work has also highlighted that erythrocytes from individuals with a severe vitamin C deficiency are fragile and prone to lysis [24], which may have affected the accuracy of our assay in deficient individuals.

## 5. Conclusions

In this study, we provide valuable data that contribute to the understanding of erythrocyte ascorbate accumulation and pharmacokinetics. These cells are often ignored with regard to vitamin C; however, they represent a substantial pool of ascorbate in the body, making up 40%–50% of blood volume. Further work investigating erythrocyte ascorbate concentrations in acute and chronically ill cohorts, and particularly those who are receiving intravenous vitamin C infusions, is warranted. Such studies would help clarify whether erythrocyte ascorbate could be useful as an indicator of steady-state plasma ascorbate concentrations in non-fasting individuals.

## References

[1] Smirnoff N. (2018). Ascorbic acid metabolism and functions: A comparison of plants and mammals. Free. Radic. Boil. Med..

[2] Khalife R., Grieco A., Khamisa K., Tinmouh A., McCudden C., Saidenberg E. (2019). Scurvy, an old story in a new time: The hematologist’s experience. Blood Cells Mol. Dis..

[3] Padayatty S.J., Levine M. (2016). Vitamin C: The known and the unknown and Goldilocks. Oral Dis..

[4] Pearson J.F., Pullar J.M., Wilson R., Spittlehouse J.K., Vissers M.C.M., Skidmore P.M.L., Willis J., Cameron V.A., Carr A.C. (2017). Vitamin C Status Correlates with Markers of Metabolic and Cognitive Health in 50-Year-Olds: Findings of the CHALICE Cohort Study. Nutrients.

[5] Schleicher R.L., Carroll M.D., Ford E.S., Lacher D.A. (2009). Serum vitamin C and the prevalence of vitamin C deficiency in the United States: 2003–2004 National Health and Nutrition Examination Survey (NHANES). Am. J. Clin. Nutr..

[6] Levine M., Conry-Cantilena C., Wang Y., Welch R.W., Washko P.W., Dhariwal K.R., Park J.B., Lazarev A., Graumlich J.F., King J. (1996). Vitamin C pharmacokinetics in healthy volunteers: Evidence for a recommended dietary allowance. Proc. Natl. Acad. Sci. USA.

[7] Wilson J.X. (2005). Regulation of Vitamin C Transport. Annu. Rev. Nutr..

[8] Savini I., Rossi A., Pierro C., Avigliano L., Catani M.V. (2008). SVCT1 and SVCT2: Key proteins for vitamin C uptake. Amino Acids.

[9] Du J., Cullen J.J., Buettner G.R. (2012). Ascorbic acid: Chemistry, biology and the treatment of cancer. Biochim. Biophys. Acta Bioenerg..

[10] Sotiriou S., Gispert S., Cheng J., Wang Y., Chen A., Hoogstraten-Miller S., Miller G.F., Kwon O., Levine M., Guttentag S.H. (2002). Ascorbic-acid transporter Slc23a1 is essential for vitamin C transport into the brain and for perinatal survival. Nat. Med..

[11] Corpe C.P., Tu H., Eck P., Wang J., Faulhaber-Walter R., Schnermann J., Margolis S., Padayatty S., Sun H., Wang Y. (2010). Vitamin C transporter Slc23a1 links renal reabsorption, vitamin C tissue accumulation, and perinatal survival in mice. J. Clin. Investig..

[12] Vera J.C., Rivas C.I., Fischbarg J., Golde D.W. (1993). Mammalian facilitative hexose transporters mediate the transport of dehydroascorbic acid. Nature.

[13] Rumsey S.C., Kwon O., Xu G.W., Burant C., Simpson I., Levine M. (1997). Glucose Transporter Isoforms GLUT1 and GLUT3 Transport Dehydroascorbic Acid. J. Boil. Chem..

[14] Mendiratta S., Qu Z.-C., May J.M. (1998). Erythrocyte Ascorbate Recycling: Antioxidant Effects in Blood. Free. Radic. Boil. Med..

[15] May J.M., Qu Z.C., Qiao H., Koury M.J. (2007). Maturational loss of the vitamin C transporter in erythrocytes. Biochem. Biophys. Res. Commun..

[16] May J.M. (1998). Ascorbate function and metabolism in the human erythrocyte. Front. Biosci..

[17] Tu H., Wang Y., Li H., Brinster L.R., Levine M. (2017). Chemical Transport Knockout for Oxidized Vitamin C, Dehydroascorbic Acid, Reveals Its Functions in vivo. EBioMedicine.

[18] Sage J.M., Carruthers A. (2014). Human erythrocytes transport dehydroascorbic acid and sugars using the same transporter complex. Am. J. Physiol. Physiol..

[19] Williamson D., Winterbourn C.C. (1980). Effect of oral administration of ascorbate on acetylphenylhydrazine-induced Heinz body formation. Br. J. Haematol..

[20] Evans R.M., Currie L., Campbell A. (1982). The distribution of ascorbic acid between various cellular components of blood, in normal individuals, and its relation to the plasma concentration. Br. J. Nutr..

[21] Li H., Tu H., Wang Y., Levine M. (2012). Vitamin C in mouse and human red blood cells: An HPLC assay. Anal. Biochem..

[22] Pullar J.M., Bayer S., Carr A.C. (2018). Appropriate Handling, Processing and Analysis of Blood Samples Is Essential to Avoid Oxidation of Vitamin C to Dehydroascorbic Acid. Antioxidants.

[23] Levine M., Wang Y., Padayatty S.J., Morrow J. (2001). A new recommended dietary allowance of vitamin C for healthy young women. Proc. Natl. Acad. Sci. USA.

[24] Tu H., Li H., Wang Y., Niyyati M., Wang Y., Leshin J., Levine M. (2015). Low Red Blood Cell Vitamin C Concentrations Induce Red Blood Cell Fragility: A Link to Diabetes Via Glucose, Glucose Transporters, and Dehydroascorbic Acid. EBioMedicine.

